# Ultrasound-Controlled Prodrug Activation: Emerging Strategies in Polymer Mechanochemistry and Sonodynamic Therapy

**DOI:** 10.1021/acsabm.4c00150

**Published:** 2024-05-02

**Authors:** Xuancheng Fu, Xiaoran Hu

**Affiliations:** † Department of Chemistry, BioInspired Institute, Syracuse University, Syracuse, New York 13244, United States

**Keywords:** ultrasound, prodrugs, polymer mechanochemistry, sonodynamic therapy, mechanophores, sonosensitizers, sonochemistry, drug delivery

## Abstract

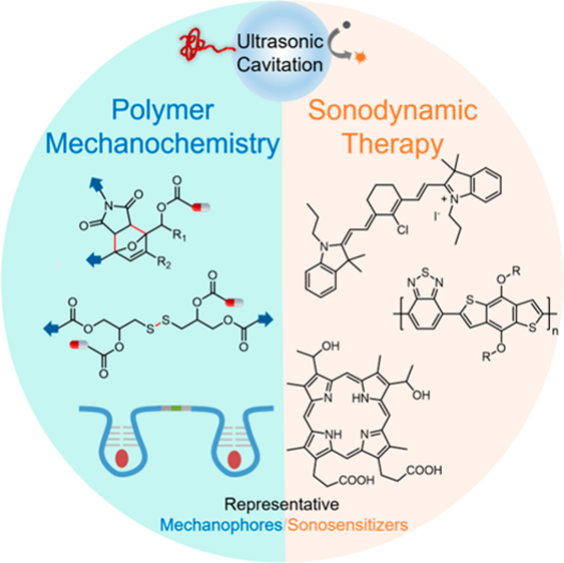

Ultrasound has gained prominence in biomedical applications due to its noninvasive nature and ability to penetrate deep tissue with spatial and temporal resolution. The burgeoning field of ultrasound-responsive prodrug systems exploits the mechanical and chemical effects of ultrasonication for the controlled activation of prodrugs. In polymer mechanochemistry, materials scientists exploit the sonomechanical effect of acoustic cavitation to mechanochemically activate force-sensitive prodrugs. On the other hand, researchers in the field of sonodynamic therapy adopt fundamentally distinct methodologies, utilizing the sonochemical effect (e.g., generation of reactive oxygen species) of ultrasound in the presence of sonosensitizers to induce chemical transformations that activate prodrugs. This cross-disciplinary review comprehensively examines these two divergent yet interrelated approaches, both of which originated from acoustic cavitation. It highlights molecular and materials design strategies and potential applications in diverse therapeutic contexts, from chemotherapy to immunotherapy and gene therapy methods, and discusses future directions in this rapidly advancing domain.

## 1 Introduction

A prodrug is a pharmacologically dormant compound that, upon administration, transforms enzymatically or chemically to release active therapeutic agents at the desired site of action. This balance between dormancy and on-demand activation ensures therapeutic precision and minimizes systemic toxicity. Since its first introduction in 1958,^1^ the prodrug concept has paved the way for tailored drug delivery systems spanning diverse fields from cancer treatments to antiviral therapeutics. Notably, physical stimuli-responsive prodrugs, especially those regulated by external stimuli such as light and ultrasound, have attracted significant attention due to their real-time control over drug activation with spatiotemporal precision.^2,3^ Ultrasound refers to mechanical sound waves beyond human hearing (20 kHz to MHz range). In comparison to light-based therapies, a notable advantage of ultrasound as a stimulus is its ability to penetrate tissues without the requirement for optical transparency in the medium, rendering ultrasound advantageous for deep tissue imaging, diagnostics, and therapeutic interventions.^4,5^

**Figure 1:**
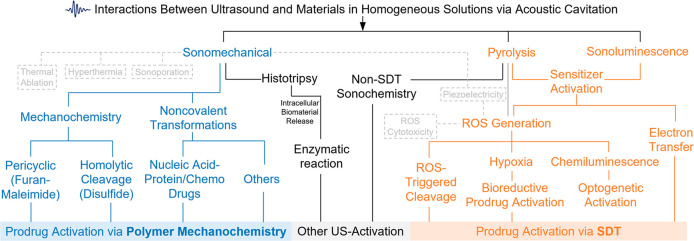
Overview of representative ultrasound–material interaction mechanisms involved in ultrasound-responsive prodrug systems: Section 2 on polymer mechanochemistry strategies, Section 3 on sonodynamic therapy (SDT) strategies, and Section 4 on additional ultrasound-mediated strategies. Texts in gray with dotted outlines represent topics mentioned in this review but not extensively covered.

In this review, we discuss recent progress in ultrasound-responsive prodrug systems divided mainly into two interrelated categories based on distinct mechanisms of ultrasound-material interactions (Figure 1): (1) polymer mechanochemistry approaches exploit acoustic cavitation-induced solvodynamic shear force to trigger the mechanochemical transformation of force-responsive molecules known as mechanophores, leading to preprogrammed structural rearrangement and release of “unmasked” functional cargo molecules. On the other hand, (2) sonodynamic therapy (SDT) strategies center on acoustic waves’ sonochemical effects, primarily the ultrasonic generation of reactive oxygen species (ROS), to initiate a cascade of chemical and biological events for prodrug activation. This review aims to foster cross-disciplinary collaborative efforts toward the development of ultrasound-responsive prodrug systems for more effective and safer therapeutics.

## 2 Polymer Mechanochemistry Approaches toward Prodrug Activation

Last two decades of research in polymer mechanochemistry have provided a library of stress-sensitive molecules known as mechanophores.^6,7^ These mechanophores exhibit mechanochemical activity that arises from a force-induced modification of their potential energy surface. Applying sufficient force lowers the barrier for specific chemical reactions, thus permitting the occurrence of these reactions within the experimental time scales.^8^ This area of research has afforded a variety of mechanophores that exhibit different functional responses to mechanical stress, such as color changes,^9,10^ catalysis activation,^11,12^ self-healing,^13,14^ and the release of functional cargo molecules.^15−18^ In this section, we discuss molecular-releasing mechanophores that are most relevant to prodrug design and therapeutic applications. Related reviews have been published on polymer mechanochemistry approaches toward ultrasound-controlled drug delivery.^19−21^ For a more extensive exploration of molecular-releasing mechanophores beyond the therapeutic context, we direct readers to recent reviews.^22−24^ Further, comprehensive review articles are available for the broader subject of polymer mechanochemistry.^6,7,25,26^

### 2.1 Ultrasound-Mediated Polymer Mechanochemistry

**Figure 2:**
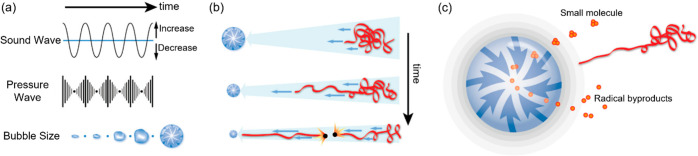
Ultrasound-induced effects in solutions are primarily from acoustic cavitation: (a) Acoustic field causes pressure variation in the solution and results in acoustic cavitation (the formation, growth, and collapse of bubbles). (b) Rapidly collapsing cavitation bubbles generate rapid liquid flows, transducing mechanical forces to the backbone of polymers near the implosion bubbles. (c) The high-temperature cavitation environment causes small molecules to form radical byproducts, while there is no evidence that the extreme conditions found in cavitation bubbles contribute to polymer degradation in nonaqueous liquids because the polymer chains have negligible vapor pressure and are unlikely to be found at the bubble interface.^27^ In aqueous solutions, pyrolysis can occur to hydrophobic polymers concentrate at the bubble–air interface. Reproduced with permission from ref (25). Copyright 2009, American Chemical Society.

In polymer mechanochemistry, solution-phase ultrasonication is a highly effective and commonly used method for studying the activation of mechanophores. Ultrasound acoustic waves cause pressure variation in the solution, leading to the generation of rapidly collapsing cavitation (i.e., the nucleation, growth, and implosive collapse of bubbles in solution).^25,27−30^ When a polymer molecule in solution is near a collapsing bubble, it is pulled toward the cavity of the bubble (Figure 2). The solvodynamic shear elongates the polymer backbone at high strain rates and thereby transduces force to mechanophore(s) covalently embedded in the polymer backbone. Notably, the force exerted is maximized near the center of the polymer chain, and longer chains experience greater forces.^31,32^ Mechanophores are typically incorporated near the center of the polymer chain or as repeating units distributed along the polymer backbone.

The rate of mechanical activation during solution-phase ultrasonication depends on both experimental factors (such as temperature, solvent, and sonication intensity) and the structure of polymers (molecular weight, chemical composition, polymer architecture, etc.).^9,27,31^ Generally, the mechanical activation rates are faster for longer polymers, at lower temperatures, in more dilute solutions, and in solvents with low volatility. This pattern aligns with these parameters’ effects on acoustic cavitation. Sonication at higher temperatures or in volatile solvents results in more solvent vapor in the bubble, cushioning the collapse and making it less violent. In dilute solutions, the polymer chains are not entangled and are free to move in the flow fields around the bubbles. Mechanical activation is also more efficient at high ultrasound intensities, due to the greater number of bubbles with larger radii.^25,27^

In a typical solution-phase ultrasonication experiment, as commonly conducted in polymer mechanochemistry research, a dilute polymer solution is subjected to high-intensity (∼ 10 W/cm^2^), low-frequency (20 kHz) pulsed ultrasound. These ultrasonication conditions are similar to those employed in ultrasonic cell lysis and are often considered unsuitable for mammalian cells. Unless specified otherwise, all experiments discussed in Section 2 of this review adhere to these standard ultrasound conditions. A growing area of research is the integration of mechanophore activation and drug release with biomedical ultrasound conditions, to harness the material development progress made in polymer mechanochemistry and apply it to improve drug delivery systems.^33−35^

### 2.2 Furan-Maleimide Mechanophores

**Figure 3:**
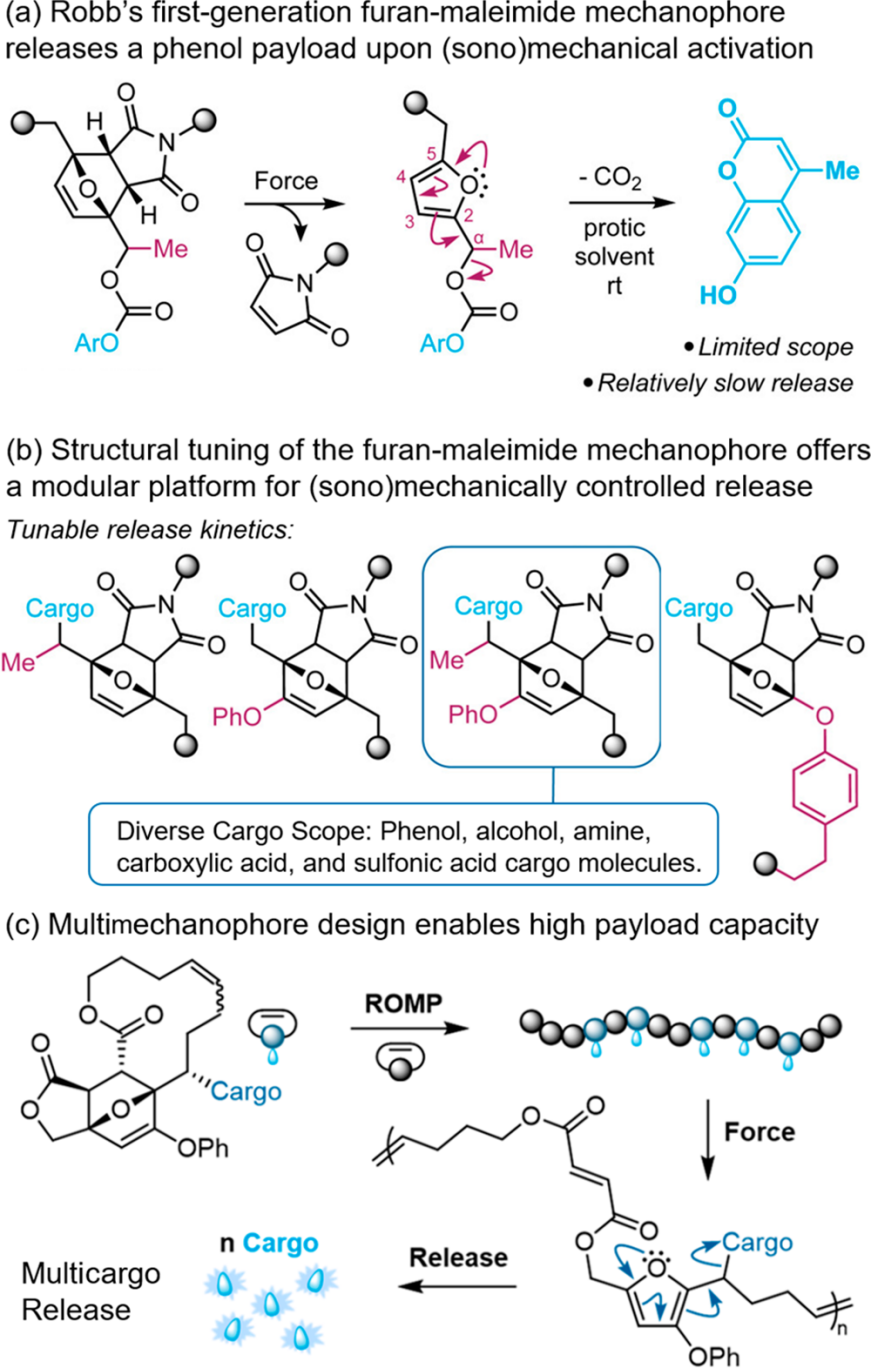
(a) (Sono)mechanochemical activation of furan-maleimide mechanophores triggers the release of payload molecules via a retro-Diels–Alder/fragmentation cascade. (b) Structural modification offers a library of furan-maleimide mechanophores capable of releasing cargo molecules bearing different functional groups. (c) A multimechanophore polymer design incorporating a nonscissile mechanophore enables (sono)mechanically triggered release of hundreds of cargo molecules per chain. Adapted with permission from refs (22 and 40). Copyright 2021, American Chemical Society. Copyright 2024, American Chemical Society.

Among mechanophores critical to the concept of ultrasound-responsive prodrugs, a notable example is the furan-maleimide mechanophore platform pioneered by the Robb group.^22^ Their original mechanophore design (Figure 3a) employed a furan–maleimide Diels–Alder adduct which under solution-phase ultrasound irradiation undergoes a mechanochemical retro-[4+2] cycloaddition reaction.^15^ This force-promoted chemical transformation unveils a latent furfuryl carbonate, which subsequently decomposes to release a covalently attached phenol molecule. A fluorogenic hydroxycoumarin served as a model payload for facile characterization of molecular release. In their subsequent studies, Robb and colleagues constructed a library of structurally diverse furan-maleimide mechanophores (Figure 3b) and broadened the range of possible cargo types, including alcohol, phenol, alkylamine, arylamine, carboxylic acid, and sulfonic molecules.^36−39^ These initial studies established a general platform that can be extended for ultrasound-controlled prodrug activation, leveraging the mechanical impact of ultrasound waves. However, the 20-kHz ultrasonication conditions used in these standard mechanochemistry experiments are generally considered as not suitable for biomedical applications.

**Figure 4:**
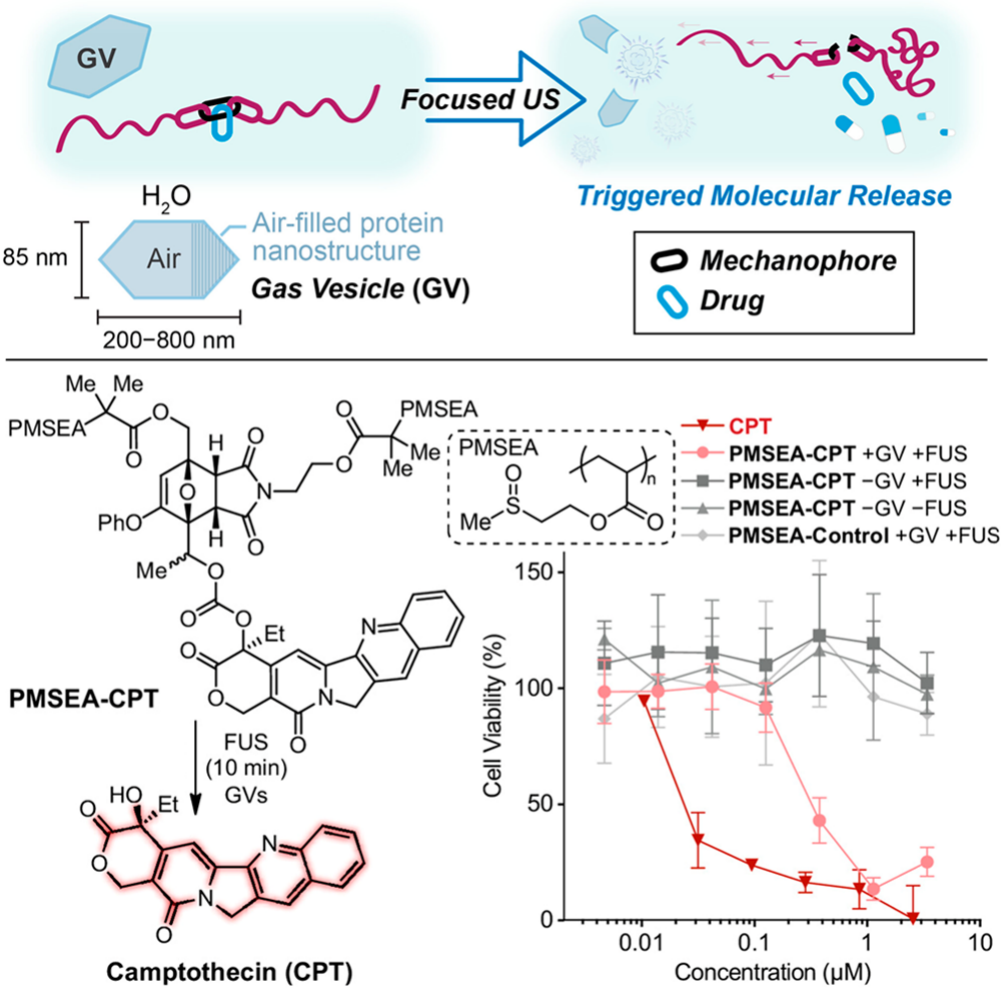
Gas vesicles (GVs)-mediated sonomechanical activation of a CPT-releasing mechanoresponsive PMSEA polymer released the anticancer drug CPT, which exhibited expected cytotoxicity to cancer cells. Sonomechanical activation was performed under physiological conditions using biocompatible focused ultrasound at 330 kHz. Adapted with permission from ref (33). Copyright 2023, The National Academy of Sciences.

Robb and Shapiro recently introduced a novel platform that synergistically couples the mechanochemical activation of mechanophores with biocompatible focused ultrasound (330 kHz) by utilizing gas vesicles (GVs) as acoustic-mechanical transducers (Figure 4).^33^ GVs are genetically encodable, pressure-sensitive protein nanostructures filled with air, typically measuring ∼ 100 nm in diameter and ∼ 500 nm in length. In this synergistic platform, GVs served as seeds for bubble formation and cavitation under the influence of biocompatible ultrasound. This resulted in amplified mechanical effect of ultrasound, effectively mediating the mechanochemical activation of molecular releasing mechanophores. As a proof-of-concept demonstration, a furan-maleimide mechanophore covalently loaded with a camptothecin (CPT) cargo molecule—essentially a mechanoresponsive CPT-prodrug—was successfully activated using focused ultrasound in the presence of GVs, which led to the expected cytotoxicity on Raji cells in vitro.

Another limitation of this mechanoresponsive prodrug strategy is the intrinsically low drug loading capacity, constrained by the macromolecular architecture where each macromolecule typically contains a single chain-centered mechanophore. There exists a threshold molecular weight, usually in the range of several tens of thousands kg/mol for polymers like poly(methyl acrylate) and other linear polymers, below which the solvodynamic shear forces transduced to the polymer chains are insufficient to initiate the mechanochemical reaction.^27,31^ The hydrophilic PMSEA polymers (Mn around 260–319 kg/mol) reported by Robb and Shapiro exemplify this limitation, exhibiting a drug loading capacity of only about 0.1 w.t.%. To overcome this limitation, Robb and co-workers employed a multiple-mechanophores macromolecular architecture^41^ that significantly enhanced the capacity for sonomechanically triggered molecular release (Figure 3c).^40^ Importantly, Robb and co-workers’ approach of coupling GV with the activation of mechanoresponsive prodrugs,^33^ along with their multimechanophore molecular release strategy,^40^ provide elegant solutions to overcome the inherent limitations of not only their furan-maleimide mechanophore platform but also other sonomechanical prodrug systems in general. These remarkable advancements pave the way for the practical application of polymer mechanochemistry in the realm of therapeutic drug delivery, showcasing its potential for translational impact.

### 2.3 Disulfide Mechanophores

**Figure 5:**
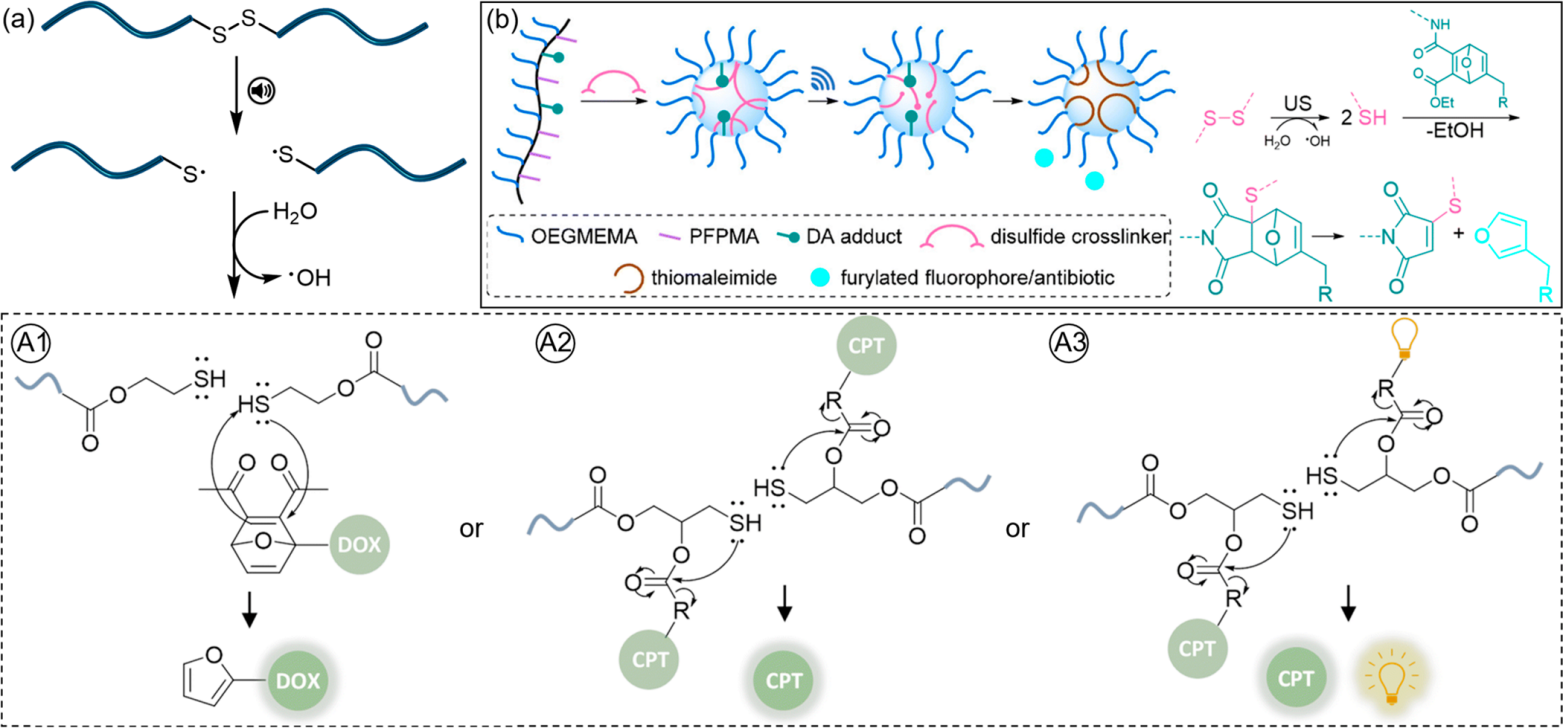
(a) Ultrasound-induced scission of disulfide-centered polymers generated thiols that subsequently triggered the release of drug or reporting molecules. Thiol intermediates underwent (A1) intermolecular Michael addition to initiate a retro Diels–Alder reaction and release the furylated doxorubicin payload,^42^ (A2) intramolecular 5-exo-trig cyclization to release CPT,^16^ and (A3) intramolecular 5-exo-trig cyclization to release CPT and fluorescent umbelliferone simultaneously.^43^ (b) Ultrasound mechanochemically induced the cleavage of disulfide cross-linkers in microgels, resulting in a Michael addition to a DA adduct that released a furan dansyl or trimethoprim.^44^ Adapted with permission from refs (19 and 44). Copyright 2022, Royal Society of Chemistry. Copyright 2022, John Wiley and Sons.

Göstl and Herrmann groups established another elegant disulfide mechanophore system for ultrasound-triggered prodrug activation (Figure 5). Disulfide bonds, being about 40% weaker than C–C bonds (dissociation energies of around 350 kJ mol^–1^), can be mechanically broken apart into thiyl radicals. These radicals then abstract hydrogen from water to form thiols, initiating cascade chemical transformations that result in the release of cargo molecules. In their pioneering study, a disulfide moiety was incorporated near the center of a water-soluble polymer (Mn = 48.9 kDa).^42^ Utilizing 20 kHz ultrasound, they demonstrated that polymer chains selectively break at the disulfide site, triggering the formation of thiols. These thiols then participate in an intermolecular Michael-addition (Figure 5A1), leading to a retro Diels–Alder reaction that liberates furylated molecules such as the drugs furosemide as well as furylated doxorubicin. In vitro experiments demonstrated that the sonicated doxorubicin-mechanophore mixture was more effective than the inactive prodrug in killing HeLa cells. However, this initial disulfide-based system faces two main limitations: (1) the inherent instability of the disulfide bond in biological redox conditions, which might cause premature leakage of the drug, and (2) the limitation of the mechanically triggered release to furan-incorporated structures.

An improved design overcomes the limitations of the first-generation disulfide system (Figure 5A2):^16,45^ drugs are covalently attached to the β-position of the disulfide moiety through a carbonate linker. Together with POEGMEA polymer attachment, the β-substitution shields the disulfide and renders it resistant to bioreduction. When exposed to 20 kHz ultrasound irradiation, mechanically generated thiols initiate an intramolecular 5-exo-trig cyclization, effectively releasing the attached CPT drug molecule. In vitro cell proliferation assays demonstrated ultrasound-induced cytotoxicity as a result of the sono-mechanochemical liberation of CPT. Further, the bifunctional character of this platform supports a theranostic approach (Figure 5A3), simultaneously activating a CPT prodrug and a fluorescent reporter umbelliferone, enabling real-time tracking of drug release and biodistribution.^43^

Herrmann and Göstl pursued a nanoparticle-based approach (Figure 5b) to alleviate the adverse effects associated with the harsh ultrasound conditions typically used in polymer mechanochemistry.^44,46,47^ While their method still involves biologically disruptive ultrasound, they innovatively integrated disulfide motifs in the cross-linkers of a microgel. This material architecture more effectively harnesses sonomechanical forces for activating mechanophores, thereby reducing the required ultrasound dosage and minimizing side effects. Five minutes of sonication led to 42% release of a fluorescent furylated antibiotic molecule (trimethoprim) from the microgel, in contrast to linear mechanophore-centered chains that require multiple hours to activate comparable amounts of mechanophores. To further explore the impact of polymer topology on the activation of mechanophores, Göstl and Herrmann recently studied the activation of disulfide mechanophores covalently embedded as cross-linkers in the cross-linked polymer shell of microbubbles.^48^ It was found that the mechanochemical activation of the disulfides is greatly accelerated using microbubbles consisting of an N_2_ core compared to commensurate solid core particles or capsules filled with liquid. These developments underscore the possibility of increasing activation efficiency and reducing adverse effects by altering the material structure, a strategy complementary to Robb and Shapiro’s use of GVs and biomedical ultrasound.^33^

### 2.4 Supramolecular Systems

**Figure 6:**
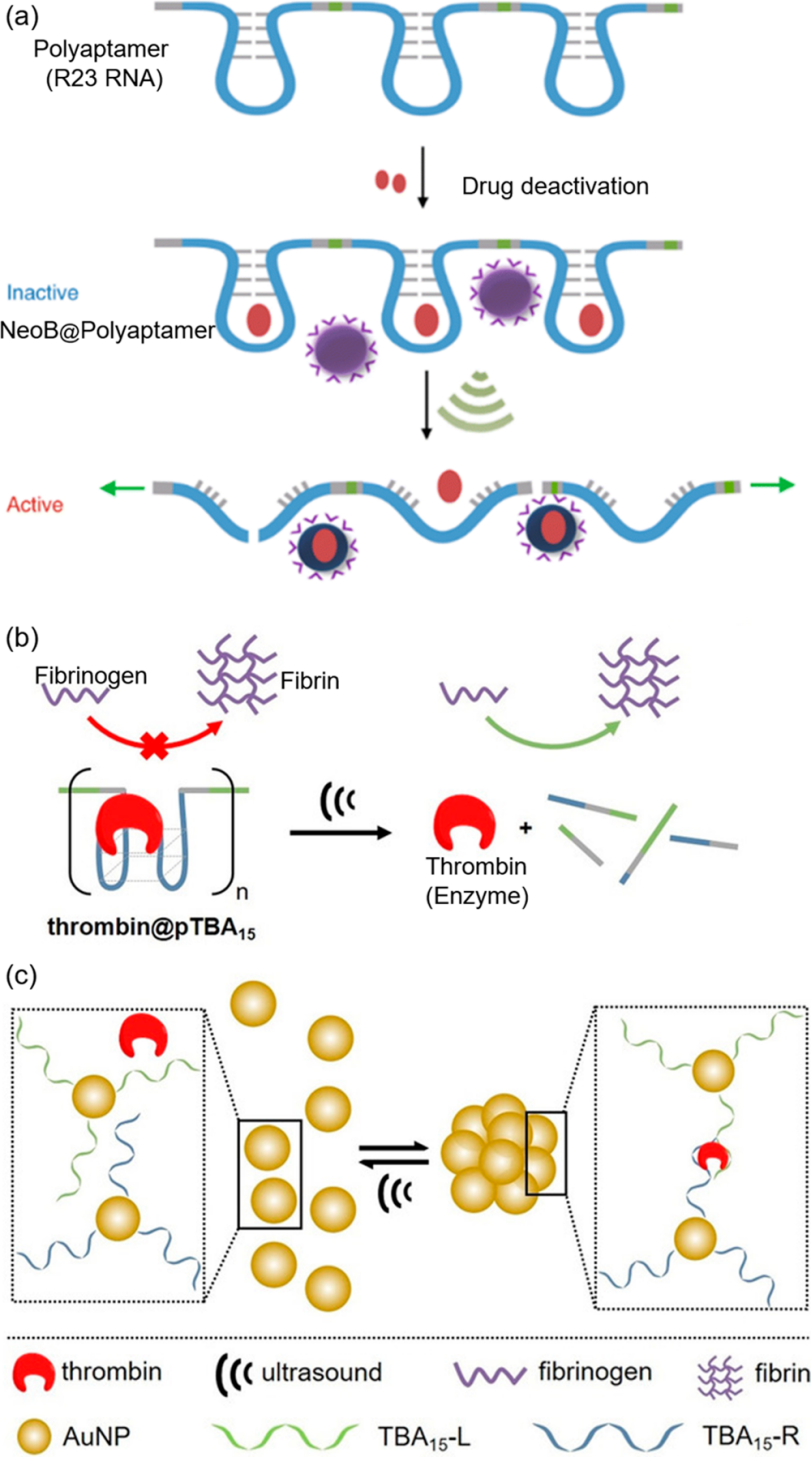
(a) NeoB was loaded into polyaptamers, inhibiting its biological activity. Sonomechanical stretching and scission released NeoB, activating its antibiotic activity.^16^ (b) From an enzyme-aptamer complex thrombin@pTBA15, ultrasound triggered the release of thrombin which catalyzed fibrinogen formation from fibrin.^49^ (c) Ultrasound treatment induced the disassembly of AuNP/aptamer/thrombin aggregates, leading to the release of active thrombin.^49^ Adapted with permission from refs (16, 19, and 49). Copyright 2021, Springer Nature. Copyright 2022, Royal Society of Chemistry. Copyright 2021, John Wiley and Sons.

In addition to their work on covalent disulfide mechanochemistry, the research groups of Herrman and Göstl have also pioneered mechanoresponsive prodrug systems based on host–guest interactions involving nucleic acid materials. In the first such system (Figure 6a), they leveraged the known binding interaction between R23 RNA aptamers and the aminoglycoside antibiotic neomycin B (NeoB).^16^ By incorporating NeoB into both individual aptamers and high molecular weight polyaptamers, they effectively inhibited its biological activity. Upon application of 20 kHz solution-phase ultrasonication, the bioinactive NeoB@Polyaptamer complex released up to 80% of the NeoB. This release was attributed to the mechanical disruption of the noncovalent host–guest interactions, subsequently reactivating NeoB’s antibiotic properties. The ultrasound-triggered release of NeoB was found to be effective in exterminating S. aureus bacteria. In contrast, the NeoB@monomeric aptamer complex showed no such activation, primarily because the monomeric aptamer’s length was insufficient to generate the necessary mechanical force through its backbone.

A comparable strategy was employed to create a mechanoresponsive aptamer-enzyme complex for ultrasound-triggered activation of enzymatic activity (Figure 6b).^49^ In this system, the activity of thrombin is initially inhibited within a polyaptamer-thrombin complex. Following exposure to low-intensity focused ultrasound (LIFU) at 5 MHz, the complex releases thrombin, restoring its enzymatic function to catalyze the transformation of fibrinogen into fibrin. Additionally, the same research groups have demonstrated LIFU-triggered activation of thrombin from aptamer-nanoparticle supramolecular assemblies (Figure 6c).^49^ Thiolated split aptamers were used to functionalize gold nanoparticles (AuNPs). The presence of thrombin led to the aggregation of these nanoparticles, thereby inhibiting thrombin’s activity. LIFU irradiation disaggregated the assembly, reactivating the enzyme.

**Figure 7:**
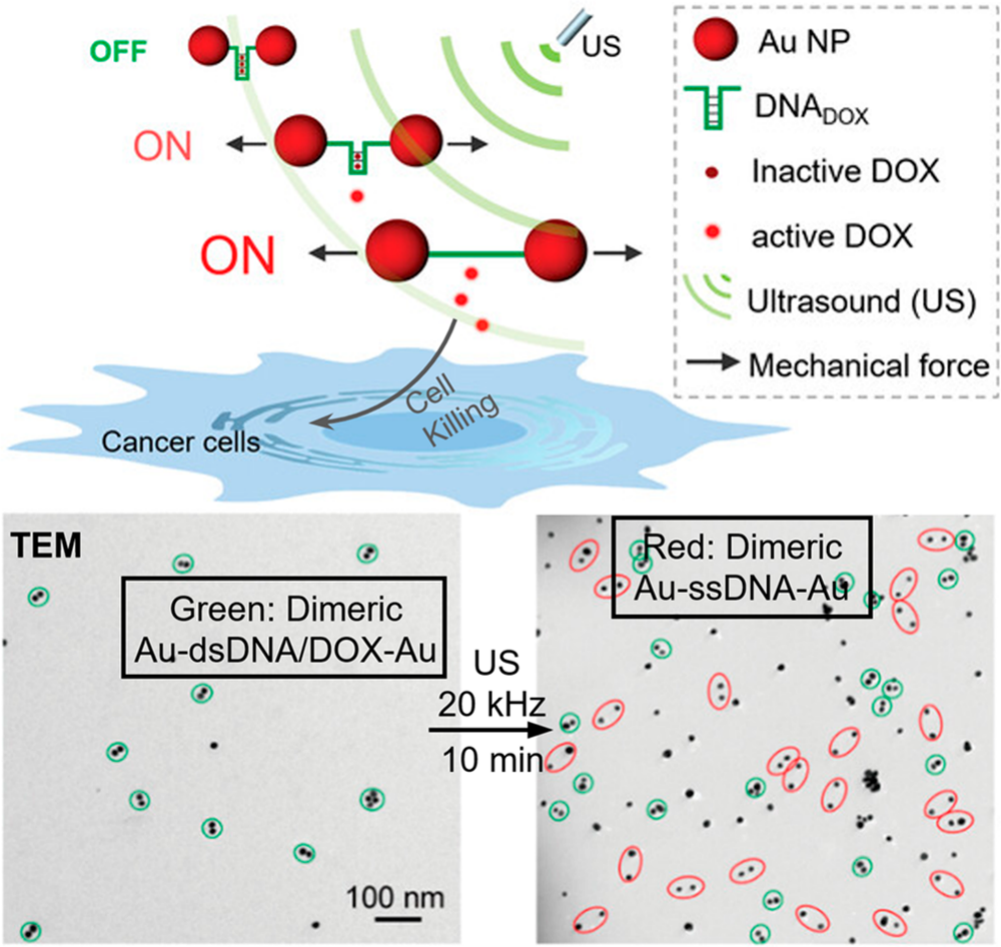
Sonomechanical force stretched the Au-DNA dimer structures to achieve DOX release. Gold nanoparticles (AuNPs) acted as a transmitter of the sonodynamic shear force, converting double-stranded DNA (the mechanophore) into single stranded. Adapted with permission from ref (50). Copyright 2022, Royal Society of Chemistry.

In a related study, Huo and Herrmann developed a mechanoresponsive DNA-nanoparticle (NP) system by linking two gold nanoparticles (AuNPs) with a specific single-stranded (ss) DNA sequence (Figure 7).^50^ This sequence was engineered to (1) hybridize into a hairpin structure, forming double-stranded (ds) DNA, and (2) allow for the noncovalent intercalation of doxorubicin (DOX), effectively rendering DOX bioinactive. Upon exposure to 20 kHz ultrasound, the dsDNA structure in the AuNP dimer was mechanically unzipped, as confirmed by transmission electron microscopy (TEM) results. This process resulted in the release of 60% of the encapsulated DOX over a 30 min sonication period. A significant decrease in cell viability was observed in LNCaP cells treated with the sonicated samples ex situ, demonstrating the successful activation of this novel mechanosensitive prodrug system.

### 2.5 Enabling Polymer Mechanochemistry under Biomedical Ultrasound

**Figure 8:**
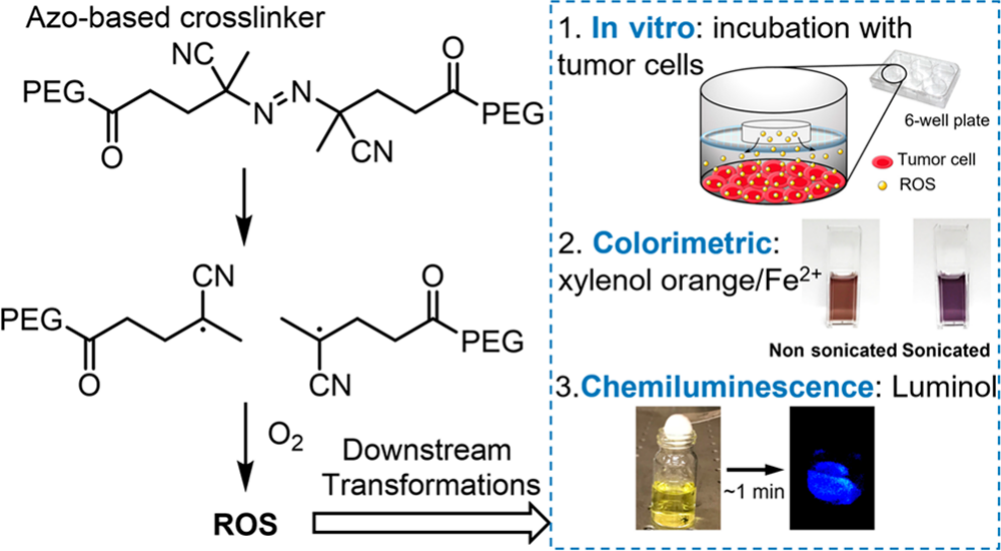
Structure of the mechanoresponsive cross-linker containing an azo group, and the sonomechanical generation free radicals and ROS from this cross-linked hydrogel. Sonomechanochemically generated ROS leads to different modes of chemical and biological responses. Adapted with permission from ref (35). Copyright 2022, The National Academy of Sciences.

An important area of development in polymer mechanochemistry is the facilitation of sono-mechanochemical activation under biomedically compatible ultrasound conditions. Beyond the recent success of Robb and Shapiro in GV-mediated mechanoresponsive prodrug activation with biocompatible focused ultrasound at 330 kHz,^33^ and the advancement of Göstl and colleagues with LIFU at 5 MHz,^49^ Li and Moore have pioneered strategies for activating mechanophores in cross-linked polymer networks using biomedical high-intensity focused ultrasound (HIFU) at 550 kHz and 1 MHz.^34^ Additionally, they have recently proposed the concept of mechanochemical dynamic therapy (MDT), where they employed ultrasound-triggered mechanical activation of azo mechanophores to produce reactive free radicals, facilitating ROS-induced cytotoxicity to cancer cells (Figure 8).^35^

### 2.6 Control Experiments Elucidate the Mechanical Origin of Mechanophore Activation

In the field of polymer mechanochemistry, the mechanical origin of mechanophore activation has been proven through control experiments. In a typical control experiment, the mechanically sensitive structure is positioned near the end of a polymer chain, and this chain-end control polymer is subjected to ultrasonic treatment. Solution-phase ultrasonication generates a shear force field that transduces force to the dissolved polymer long chains, with the applied force maximized near the center of the polymer chain. The chain-end attached mechanosensitive structures in control polymers experience minimal mechanical force, but they experience otherwise identical ultrasonic (thermal, chemical, etc.) effects as their chain-center functionalized counterparts. The absence of ultrasound-triggered mechanophore activation in these chain-end control polymers, contrasted with successful activation in polymers of similar chain length where the mechanophore is centrally located, confirms that the ultrasonic activation of chain-centered mechanophores is indeed mechanically driven. There is no evidence that ultrasound-triggered chemical transformation in polymers result from the extreme conditions of temperature found in cavitation bubbles in nonaqueous liquids, because polymer chains have negligible vapor pressure and are unlikely to be found at the bubble interface.^27^

In polymer mechanochemistry, it is a common belief that small molecules are not significantly affected by elongational or shear forces. On the other hand, the mechanisms in various SDT systems, which we discuss in the latter half of this review, are still under active investigation. Some studies cited the sonomechanical effect to explain the activation of small-molecule sonosensitizers in SDT systems,^51−54^ but the evidence supporting this mechanism is lacking. Further mechanistic studies would be necessary before attributing the ultrasonic activation of sensitizers in these systems to mechanical effects. As we will explore in the subsequent section, the activation of small-molecule sonosensitizers is generally believed to be originated from the thermal effect of collapsing cavitation (both sonoluminescence and pyrolysis are thermal in origin).^55^

## 3 Sonodynamic Therapy Approaches toward Prodrug Activation

The development of sonodynamic therapy (SDT) strategies has paralleled advances in polymer mechanochemistry, providing a number of ultrasound-responsive prodrug systems grounded in mechanisms distinct from those of polymer mechanochemistry. In 1989, Umemura and co-workers first reported the cytotoxic effects of hematoporphyrin under acoustic irradiation,^56^ and they named this method as sonodynamic therapy in 1992.^57^ Analogous to photodynamic therapy (PDT), SDT usually involves the activation of a sensitizer by external stimuli and the generation of reactive oxygen species (ROS). However, SDT utilizes ultrasound waves as the activating stimulus, unlike PDT which uses light. These ultrasound-responsive sensitizers known as sonosensitizers are nontoxic in the absence of ultrasound but become cytotoxic upon ultrasound exposure. While the term ’SDT’ is sometimes broadly used in literature to encompass ultrasound-induced therapeutic effects, such as enhanced local pharmacokinetics or drug biodistribution through sonoporation of cell membranes, it is recommended to use ’SDT’ only to describe ultrasound-dependent enhancement of the cytotoxic action of sonosensitizers.^58,59^

A primary mechanism for the therapeutic effectiveness of SDT is the chemical action of ultrasound-activated sonosensitizers, particularly in generating ROS. ROS are highly reactive molecules that can cause oxidative stress, leading to damage and apoptosis in target cells. Beyond this direct therapeutic action of ROS, an emerging strategy involves utilizing ROS to trigger downstream chemical transformations such as prodrug activation. Alongside the ROS-mediated mechanism, an alternative mechanism explaining the effect of sensitizers under ultrasound suggests that certain sonosensitizers such as porphyrin,^60−62^ Vitamin E,^63^ and Trolox,^64^ may embed into lipid membrane bilayers, thereby increasing cellular susceptibility to sonomechanical lysis under acoustic cavitation.^58^ Readers are directed to recent review papers for different aspects of SDT such as experimental parameters,^65^ mechanisms,^58,66^ and SDT-based nanomedicines.^58,67,68^

### 3.1 Mechanisms for Sonosensitizer Activation

#### 3.1.1 Excitation by Sonoluminescence

The exact mechanisms for sonosensitizer activation remain a subject of ongoing research. A primary mechanism proposed involves sonoluminescence, i.e. the emission of light from cavitating microbubbles under ultrasound irradiation.^30^ Sonoluminescence of water has a continuum spectrum in the range of 350–650 nm, and sonoluminescence has also been observed in agar gels^69^ and biological tissues.^70,71^ It is hypothesized that sonoluminescence as an energy source photochemically activates sonosensitizers to the electronic excited state. Subsequently, the excited sensitizer produces ROS in a manner similar to PDT processes: (i) the excited sensitizer can undergo electron transfer with adjacent oxygen, water, or other molecules to generate type I ROS such as free radicals •OH, O_2_^•-^; or (ii) sensitizers in their triplet state can undergo energy transfer with surrounding oxygen to produce singlet oxygen (Type II ROS). Type II ROS is commonly considered as the main mediator in SDT systems.^72,73^

Support for the sonoluminescence-mediated activation for a variety of sonosensitizers includes: (1) structural similarities between sonosensitizers and photosensitizers; (2) evidence supporting sonoluminescence-mediated activation of some sonosensitizers, such as RB,^74^ hematoporphyrin^75^ and metal-porphyrin complexes;^76^ and (3) a correlation in some sonosensitizers between the efficiency of ROS generation and their photosensitizing efficiency under white light,^77^ although this correlation is not consistently observed in terms of light- versus ultrasound-induced cytotoxicity in other groups of sensitizers.^62^ For counterpoints and findings that challenge the sonoluminescence-based mechanism, readers are referred to additional literature.^58,66^

**Figure 9:**
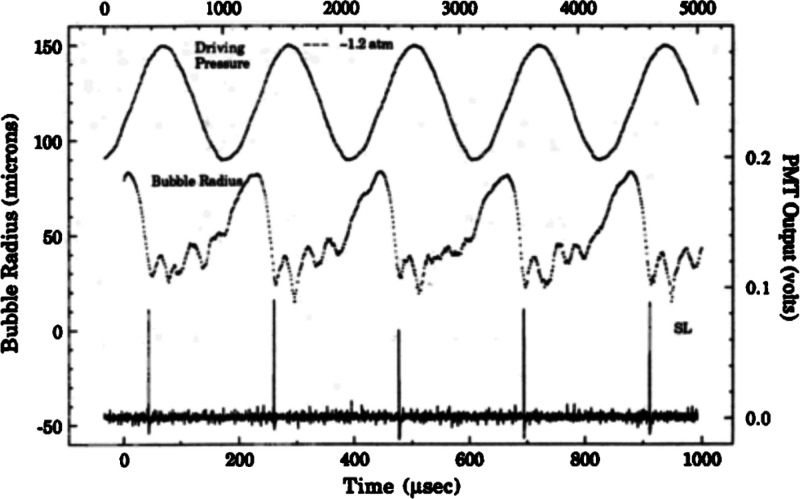
Sound field (top), bubble radius (middle), and light output (bottom) vs time for a sonoluminescencing bubble under 22.3 kHz ultrasound. Sonoluminescence occurred at the most compressed phase of bubble collapsing. Reproduced with permission from ref (81). Copyright 1992, Acoustical Society of America.

Research in the last century have concluded that sonoluminescence originates from chemical reactions of high-energy species formed during cavitation, and sonoluminescence is a form of chemiluminescence in origin.^55,78^ Historically, several hypotheses have been proposed to explain the origin of sonoluminescence: (a) the thermochemical theory, (b) the blackbody theory, (c) electrical microdischarge theory, along with less popular theories such as the mechanochemical theory, triboluminescence theory and the balloelectric theory.^79,80^ The discharge theory (c) was accepted by fairy many investigators in the early years and predicts that sonoluminescence should occur during the process of bubble growth. However, credible evidence contradicts this theory, showing that sonoluminescence happens at the collapsing phase of cavitation. For instance, Gaitan et al. reported judiciously controlled single-bubble cavitation experiments,^81^ observing that each bubble emitted a single light pulse (pulse duration <50 ps) synchronously with each acoustic cycle, as shown in Figure 9. Both the thermochemical (a) and blackbody (b) theories attribute the origin of luminescence to the thermal effect of the extremely high-temperature (∼ 5000 °C) and high-pressure (∼ 1000 atm) environment in cavitating microbubbles:^27,55,80,82^ the thermochemical theory attributes luminescence from the recombination of free ions produced through thermal dissociation, while the other theory attributes the luminescence to the blackbody radiation of hot gas molecules inside the bubbles. Verrall and Sehgal observed selective quenching of specific portions of the sonoluminescence continuum upon the addition of nitric acid.^83^ Based on Verrall and Sehgal’s findings and other evidence, Suslick and co-workers explicitly concluded that the principal source of sonoluminescence is chemiluminescence resulting from chemical reactions of high-energy species in the extreme cavitation environment, rather than blackbody radiation.^55,78,84,85^

#### 3.1.2 Activation through Pyrolysis

Pyrolysis as another cavitation-mediated thermal process has also been proposed to be responsible for the ultrasonic activation of sensitizers.^82^ The high-temperature, high-pressure cavitating bubbles serve as sonochemical reactors, facilitating the pyrolysis of molecules either inside the cavitation, at the gas–liquid interface, or within the heated shell (∼500 molecules thick).^55,82^ The sonolysis of water, in the absence of sensitizers, has been extensively studied, with H_2_ and H_2_O_2_ identified as primary products.^30,55^ Moreover, the generation of free radicals and other reactive species from water during acoustic cavitation has been evidenced, regardless of the presence or absence of a sonosensitizer.^27,80,86−89^ For example, Riesz and co-workers demonstrated definitively the generation of hydrogen atoms and hydroxy radicals during ultrasound irradiation using electron paramagnetic resonance with chemical spin traps.^82,89^

In SDT, it has been proposed that the presence of sonosensitizers amplifies the pyrolysis-mediated therapeutic effects beyond what is achieved by ultrasound alone.^82^ Sonosensitizers like Hematoporphyrin and Rose Bengal^90^ act like surfactants to accumulate at the gas–liquid interface of cavitating bubbles. Consequently, hydroxy radicals produced within collapsing cavitation bubbles preferentially engage with these nearby sensitizing surfactants over other solutes in the bulk. This engagement generates sensitizer-derived radical intermediates that can migrate the necessary distance to attack critical cellular sites.^90^ In the absence of sensitizers, sonochemically generated •OH and •H radicals atoms have short half-lives and limited diffusion distances, reacting nonselectively with organic solutes. Additionally, certain small molecules, such as azo compounds^91,92^ or solvent molecules like DMF^93,94^ and DMSO,^94^ can be broken apart to generate free radicals in the interior or around the acoustic cavitation. These radicals then interact with endogenous substances to produce ROS that are responsible for sonodynamic cell killing. While pyrolysis is a plausible mechanism for the activation of sonosensitizer and ROS generation in SDT systems, there is ongoing debate surrounding this hypothesis.^58,74^

### 3.2 Sonosensitizers

**Scheme 1:**
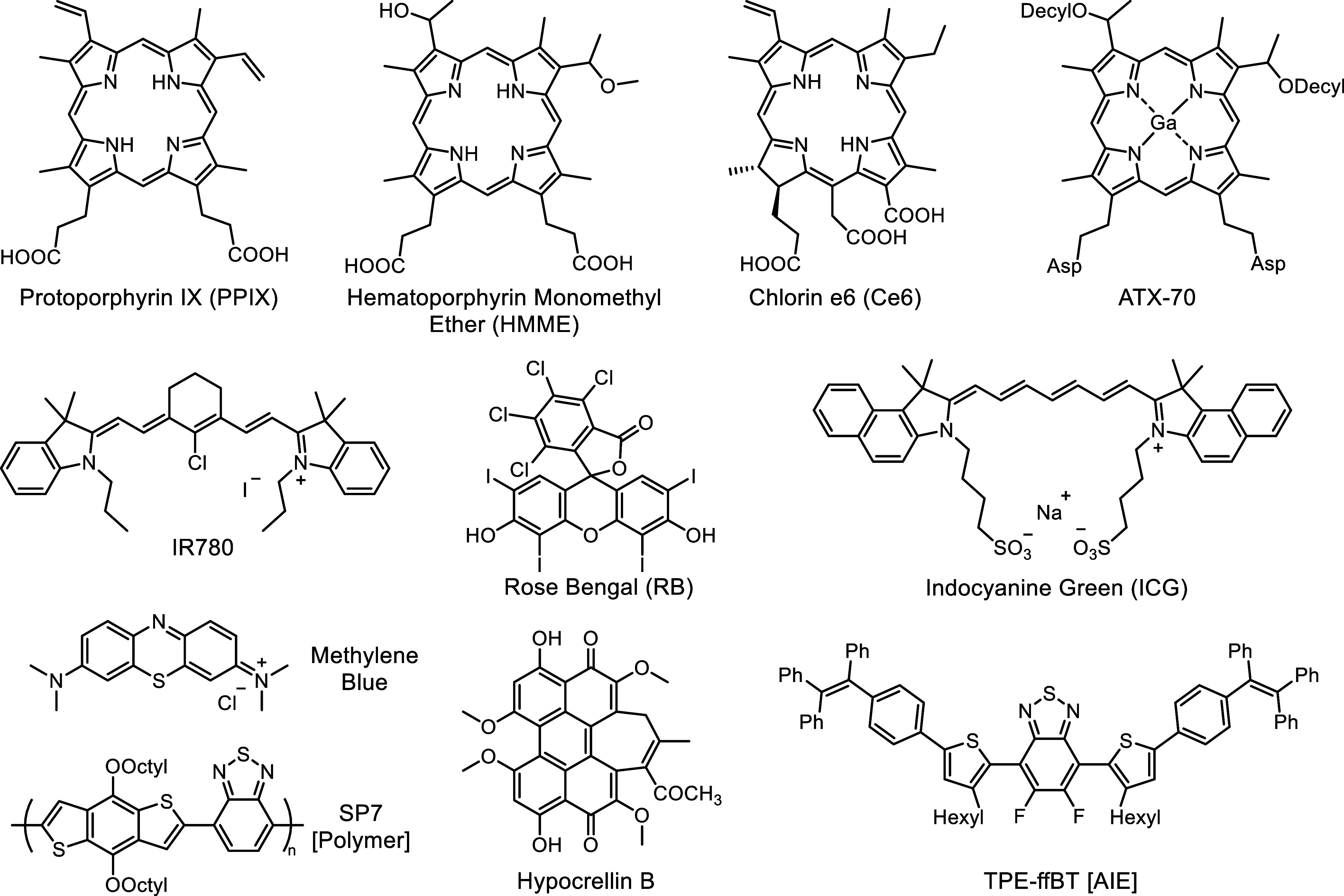
Representative Structures of Organic Sonosensitizers

Last three decades of research in SDT have led to the discovery of a wide array of sonosensitizers, including both organic (macro)molecules^95^ and inorganic nanomaterials. These sonosensitizers are nontoxic without ultrasound treatment but exhibit cell cytotoxicity through ROS production or other means. Some representative small-molecular organic sonosensitizers include (Scheme 1): (1) porphyrin derivatives such as protoporphyrin IX (PPIX),^73,96−99^ hematoporphyrin monomethyl ether (HMME),^100,101^ Chlorin e6 (Ce6),^102^ and ATX-70;^103^ (2) xanthene-based sensitizers such as Rose Bengal (RB) and its derivatives;^104,105^ (3) indocyanine-based structures such as IR-780 and indocynine green (ICG);^106^ (4) aggregation-induced emission (AIE) molecules;^107,108^ (5) conjugated polymers;^95^ (6) others compounds such as methylene blue,^109^ and hypocrellin B.^110^ Moreover, inorganic nanomaterials-based sonosensitizers such as TiO_2_ nanoparticles have also been reported to generate ROS under ultrasound irradiation.^111^ Additionally, novel piezoelectric materials have been explored to mediate the ultrasonic generation of ROS.^112^ This review will focus on organic sonosensitizer-mediated SDT approaches. For a comprehensive overview of sonosensitizers, we direct readers to recent review papers in the field.^58,113^

### 3.3 Ultrasound-Responsive Prodrugs Enabled by ROS-Cleavable Capping Groups

**Figure 10:**
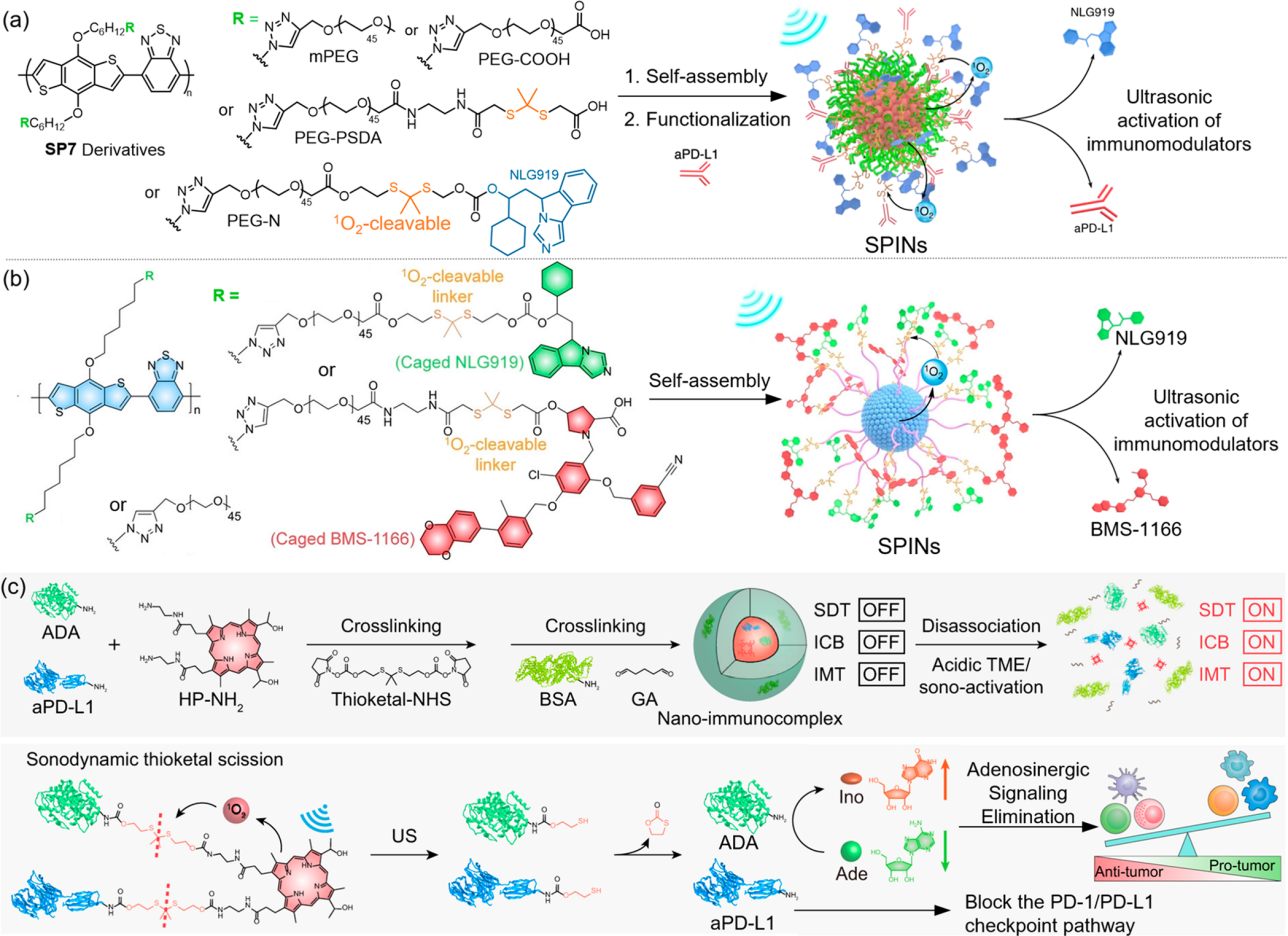
(a–c) Structures of semiconducting polymer nanoparticles developed by Pu and co-workers, and schematic illustration of immunomodulator prodrug activation mediated by ultrasound-triggered ROS generation. Adapted with permission from refs (77, 121, and 122). Copyright 2022, Nature Portfolio. Copyright 2023, John Wiley and Sons. Copyright 2022, Nature Portfolio.

A widely used strategy in designing ultrasound-responsive prodrugs involves chemically modifying drug molecules with ROS-cleavable groups, such as the thioketal group.^114−119^ Ultrasonic production of ROS triggers the thioketal cleavage followed by preprogrammed cascade structural rearrangement to release the active form of therapeutic molecules. Zhang and Pu reported a semiconducting polymer immunomodulatory nanoparticle (SPIN) comprising a sonosensitizing core made of a semiconducting polymer (SP) SP7 that efficiently generates singlet oxygen under ultrasound (Figure 10a).^77^ Immunomodulators NLG919 (a potent IDO inhibitor, enhances the proliferation of cytotoxic T lymphocytes and suppresses regulatory T cells) and antiprogrammed death-ligand 1 antibody (aPD-L1, targeting PD-L1 on cancer cells and preventing T-cell exhaustion, thereby bolstering T-cell immunity against the tumor) are conjugated to the SP7 backbone via this ROS-cleavable thioketal linker. The resulting nanoparticles effectively accumulate into tumors in vivo after systemic administration, and they induced remarkable antitumor immunity under ultrasound irradiation (1.0 MHz) through the generation of ^1^O_2_ that can (1) directly induce immunogenic cell death (ICD) in tumors, (2) reprogram the tumor microenvironment through upregulating expression levels of PD-L1 and indoleamine 2,3-dioxygenase (IDO), and (3) release immunomodulators NLG919 and aPD-L1 through ^1^O_2_-triggered thioketal cleavage. The codelivery of sonosensitizer and ^1^O_2_-responsive prodrugs of NLG919 and aPD-L1 achieved a synergetic effect, enhancing the efficacy of immunotherapy.^120^ Importantly, the immunotherapeutic activity of these two immunomodulators remains inhibited in their prodrug forms within the nanoparticles, and their ultrasound-controlled activation at targeted tumor sites greatly alleviated the systemic immune-related adverse events (irAEs).

In their subsequent research, Pu and co-workers engineered a SPIN nanoparticle comprising an analogous sonosensitizing polymer core but grafted with PEG chains (Figure 10b).^121^ Two small-molecule immunomodulators, NLG919 (*vide supra*) and BMS-1166 (blocking the PD-L1/PD-1 biding and promoting T-cell activity), are covalently linked to the PEG shell via a ^1^O_2_-cleavable thioketal. Ultrasonic (1.0 MHz) generation of ^1^O_2_ not only induces ICD in tumor cells but also initiates thioketal scission, activating the NLG919 and BMS-1166 prodrugs that synergistically boosted the antitumor immune response by reversing two tumor immunosuppressive pathways.

Pu and co-workers also employed the ^1^O_2_-cleavable thioketal linker in their design of a nanoimmunocomplex for sono-metabolic checkpoint trimodal cancer therapy (Figure 10c).^122^ This nanoimmunocomplex features all FDA-approved components, including the sonosensitizer hematoporphyrin, the checkpoint blockade inhibitor aPD-L1, the immunometabolic reprogramming enzyme adenosine deaminase (ADA), and bovine serum albumin (BSA). These elements are covalently assembled into a single nanoparticle using acid-cleavable imine and sono-activatable thioketal linkers. Under normal physiological conditions, hematoporphyrin, aPD-L1, and ADA remain inactive due to their cross-linked immobilization within the nanoimmunocomplex. Only in the concurrence of the acidic tumor microenvironment and ultrasound irradiation (1.0 MHz), the nanoimmunocomplexes are activated to (1) generate ^1^O_2_ to eliminate tumor cells and induce ICD for improved tumor immunogenicity, and (2) unleash aPD-L1 and ADA via the scission of imine and thioketal bonds. Besides the immunotherapeutic effect of aPD-L1 (*vide supra*), the ultrasound-activated ADA is a metabolic enzyme that catalytically depletes the toxic metabolites adenosine (Ade) into inosine (Ino). This further results in the elimination of adenosinergic signaling and reprogramming of the immunosuppressive tumor microenvironment, which eventually promotes the activation of antitumorigenic immune effector cells and inhibition of protumorigenic immune suppressor cells. Therefore, this nanoimmunocomplex exerts synergistic antitumor effects via the tumor-specific, ultrasound-controlled metabolic checkpoint trimodal cancer therapy approach. Additionally, Pu and co-workers have pioneered various sonodynamic immunotherapy methods that do not involve the prodrug concept.^123−126^

**Figure 11:**
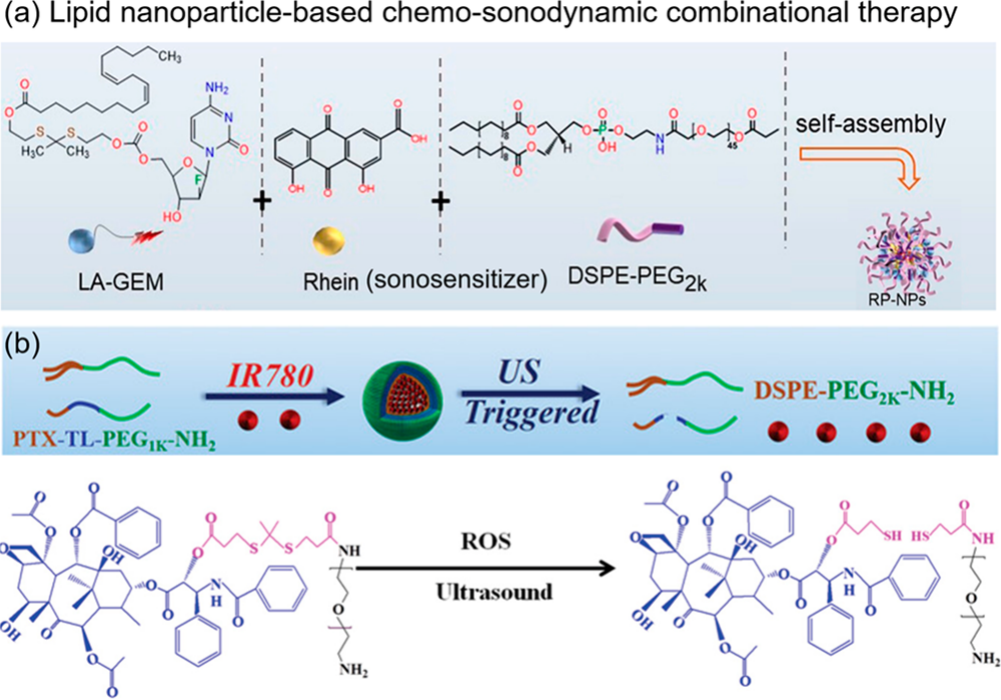
(a) The structure of a lipid nanoparticle comprising a ROS-activatable prodrug (LA-GEM), a sonosensitizer (Rhein), and a surfactant (DSPE-PEG2k). (b) The structure of an analogous nanoparticle comprising a ROS-activatable amphiphilic prodrug (PTX-TL-PEG1k-NH_2_), a sonosensitizer (IR780), and a DSPE-based surfactant. Reproduced with permission from refs (127 and 128). Copyright 2023, John Wiley and Sons. Copyright 2023, John Wiley and Sons.

The ROS-cleavable thioketal linker has also been employed by Wang and co-workers in a nanoparticle-based chemo-sonodynamic combinational therapy for the noninvasive elimination of hypoxic tumors (Figure 11a).^127^ Rhein (a natural anthraquinone known for its sonosensitizing activity) and a thioketal-capped prodrug LA-GEM were encapsulated within lipid nanoparticles. Ultrasound irradiation (7.0 MHz) excites Rhein and leads to the production of ROS, which subsequently triggers the thioketal cleavage and releases a chemotherapeutic agent gemcitabine (GEM). Ultrasonic production of ROS also disrupts redox homeostasis via mitochondrial pathways and causes damage to hypoxic tumor cells. The synergistic effect of chemo-sonodynamic combinational treatment showed notable antitumor efficacy in vitro and in vivo.

Zong and Wan reported another self-assembled nanoparticle system (Figure 11b) composed of the sonosensitizer IR780, a thioketal-capped paclitaxel (PTX) prodrug, and a DSPE-based surfactant.^128^ IR780 produces high levels of ROS under focused ultrasound (1.0 MHz) to activate the PTX prodrug via thioketal cleavage. The subsequent release of the chemotherapeutic drug PTX, combined with the inherent bioeffects of SDT, resulted in a synergistic action that effectively killed tumor cells.

### 3.4 Prodrug Activation via Electron Transfer

**Figure 12:**
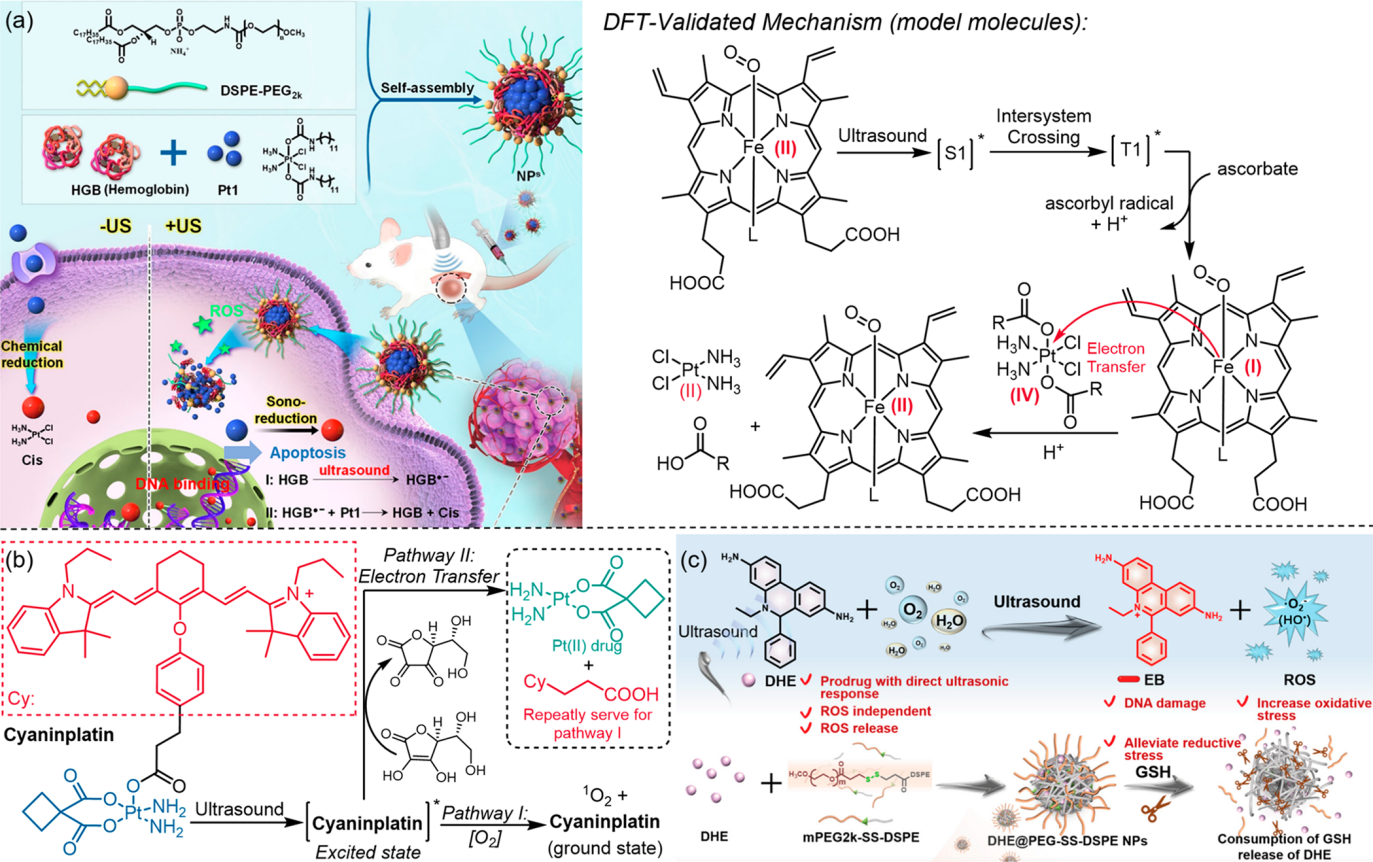
Sonodynamic activation of prodrugs through redox reactions. (a) Schematic illustration of Pt(IV) prodrug Pt1’s sonodynamic activation with a hemoglobin sonosensitizer and the proposed mechanism for the reductive activation (right side). (b) Structure of a Pt(IV) prodrug Cyaninplatin and its sonodynamic activity through two synergistic pathways. (c) DHE’s dual function as a sonosensitizer itself to generate ROS and as an activable prodrug to convert into the cytotoxic ethidium (EB) form under ultrasound irradiation. Adapted with permission from refs (51, 134, and 135). Copyright 2023, American Association for the Advancement of Science. Copyright 2023, John Wiley and Sons. Copyright 2023, John Wiley and Sons.

Photoinduced electron transfer (PET) refers to an excited-state electron transfer process and is a key process in type-I PDT.^129,130^ Direct electron transfer between excited sensitizers and prodrug structures has been widely explored to regulate prodrug activation in PDT systems.^130−133^ More recently, electron-transfer-based strategies have also been used in sonodynamic therapy (SDT) systems. For example, Xiao and Karges elegantly designed an ultrasound-responsive prodrug system that is activated through an electron-transfer mechanism (Figure 12a).^134^ They encapsulated a lipophilic Pt(IV) prodrug Pt1 and a sonosensitizer hemoglobin (HGB) into nanoparticles. The nanoparticles selectively accumulated at the mice tumor site upon intravenous injection, and the Pt(IV) prodrug was reduced to the cytotoxic cisplatin Pt(II) upon ultrasound exposure at 1 MHz, almost completely eradicating the tumor. The proposed mechanism for the reduction of the Pt(IV) prodrug, supported by density functional theory (DFT) calculations, involves the following steps: sonosensitizer are excited to an excited S1 state under ultrasound irradiation, and then converts to the excited triplet state through intersystem crossing. While energy transfer from the triplet-sate sonosensitizer to the Pt(IV) prodrug is possible, DFT calculations suggest it is more efficiently transferred to ascorbate, acting as a mediator. The spin density plot for the triplet state of the sonosensitizer concentrates on the Fe(II) center, which can accept an electron from ascorbate, thereby forming a reactive Fe(I) center. This reactive center can then further transfer an electron to the Pt(IV), resulting in the desired reduction of the prodrug into its active form.

Zhu and Wang reported a novel ultrasound-activatable (1 or 3 MHz, LIFU) Pt(IV) prodrug Cyaninplatin composed of an IR780 cyanine sonosensitizer motif covalently linked to a redox responsive Pt(IV) moiety (Figure 12b).^51^ The mechanism proposed for the ultrasonic activation of Cyaninplatin involves: (1) Acoustic cavitation excites the cyanine sensitizing motif in the Cyaninplatin; (2) The excited prodrug then transforms through two possible pathways: first, following a type II sonodynamic process to generate singlet oxygen, and second, undergoing intermolecular electron transfer from an electron donor like sodium ascorbate or nicotinamide adenine dinucleotide (NADH), reducing the prodrug to its active Pt(II) form. FUS (around 1–3 MHz) was employed to trigger the burst release of the Pt(II) drug molecule and simultaneously depleted intracellular reductants during the ultrasound-mediated reduction, leading to ROS-induced damage. This Pt(IV) prodrug demonstrated outstanding anticancer efficacy in a mouse model, and it also exhibited fluorescence turn-on and acted as a contrast agent for NIR and photoacoustic imaging.

Du introduced a unique ultrasound-responsive prodrug dihydroethidium (DHE) featuring dual functions (Figure 12c):^135^ DHE serves (1) as a sonosensitizer itself to generate ROS and (2) as an activable prodrug to convert into the cytotoxic ethidium (EB) form under ultrasound irradiation (1 MHz). DHE was encapsulated within GSH-degradable nanoparticles, enhancing drug accumulation in tumor tissues which ensures high biosafety of this nanoplatform. The synergistic dual functionality of DHE contributes to its antitumor effect, significantly inhibiting tumor growth in vivo, effectively inhibiting tumor growth, reducing the expression of metastasis-related proteins, and inhibiting lung metastasis. Mechanistically, it was proposed that (1) DHE gets excited by sonoluminescence to its excited state; (2) the excited DHE reacts with oxygen molecules in water to generate O_2_^•–^ radical and EB, and releases protons (H_3_O^+^). Next, (3) the generated O_2_^•–^ further reacts with ground-state DHE to produce EB and •OH.

Recently, Cheng, Chen and co-workers developed BiOCl–Au-Ag2S Z-scheme heterostructure nanoparticles loaded with a redox-activatable drug tirapazamine (TPZ).^136^ These nanoparticles showed efficient electron–hole separation under ultrasound irradiation and exhibited a high redox potential. The resulting redox environment activated the redox-responsive drug TPZ, which exhibited cytotoxicity to tumor both in vitro and in vivo.

**Figure 13:**
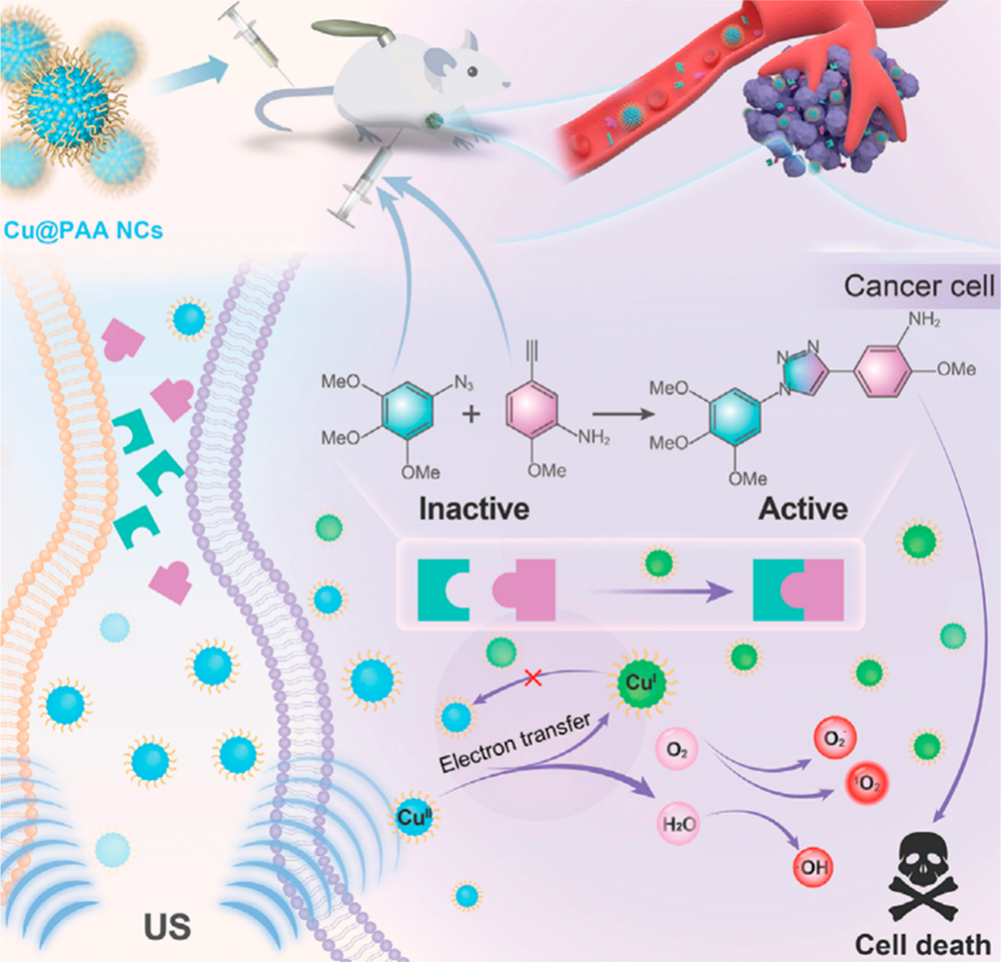
Ultrasound-controlled site-specific bioorthogonal catalytic azide–alkyne cycloaddition reaction produced a triazole drug in situ and triggered robust sonodynamic therapy. Reproduced with permission from ref (137). Copyright 2023, John Wiley and Sons.

In contrast to other approaches discussed in this section where the sonodynamic redox environment directly activated redox-responsive prodrugs, Chen, Feng, and Liu groups introduced a two-step “ultrasound-driven biorthogonal catalytic therapy” approach (Figure 13) employing a poly(acrylic acid)-modified copper nanocomplex (Cu@PAA).^137^ This novel approach (1) first harnesses the sonodynamic redox environment to reduce Cu(II) to Cu(I), and (2) uses the resulting Cu(I) as a catalyst to synthesize the triazole drug molecule, 2-methoxy-5-(1-(3,4,5-trimethoxyphenyl)-1H-1,2,3-triazol-4-yl)aniline, through a copper-catalyzed azide–alkyne cycloaddition (CuAAC) reaction between nontoxic drug precursors. The ultrasound-activated catalytical conversion of precursors into the triazole drug was successfully demonstrated in both 4T1 cells and tumor xenograft murine models.

### 3.5 Sonosensitizer Prodrug (Activatable Sonosensitizer)

**Figure 14:**
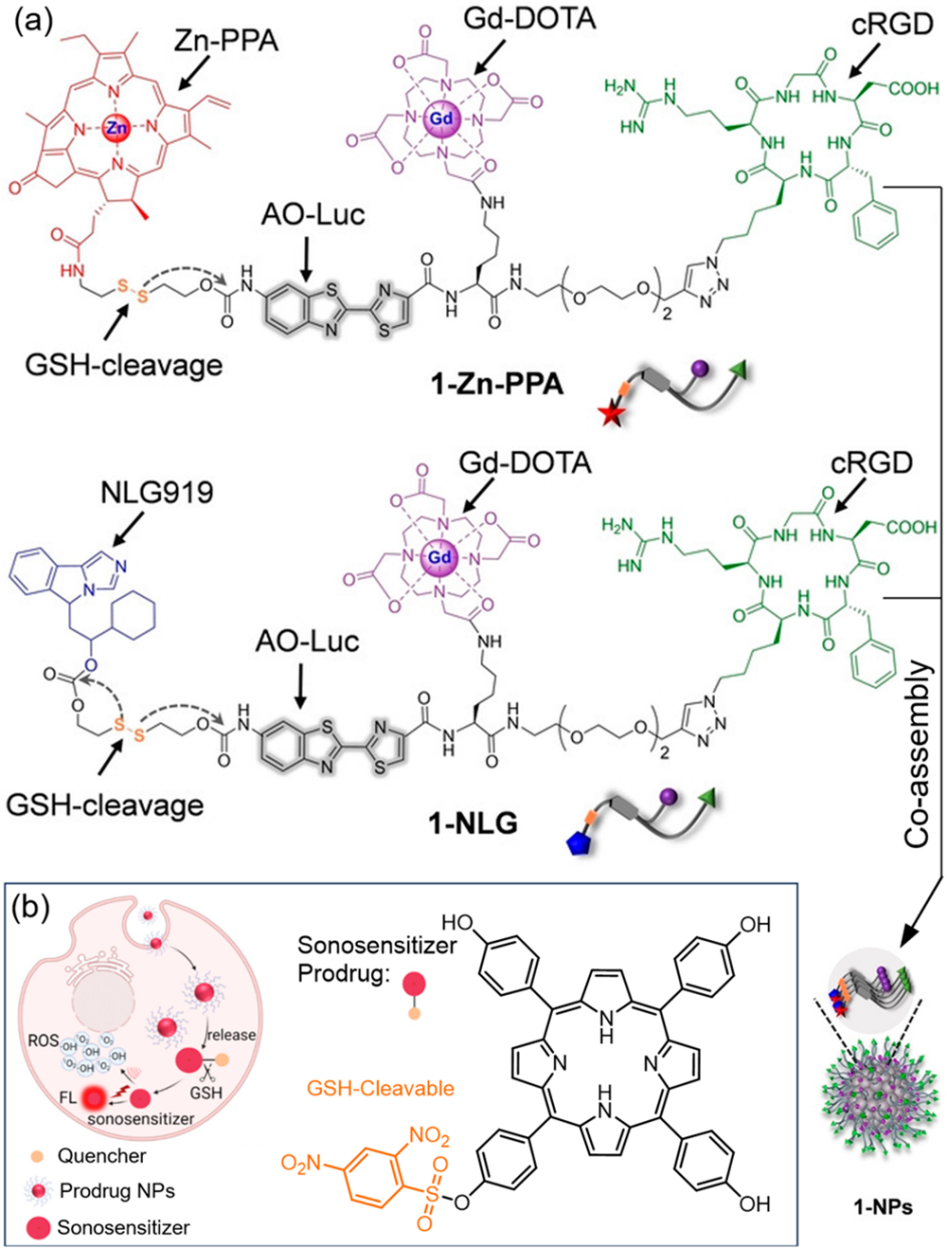
SDT systems involving GSH-activatable sonosensitizers. (a) Structures of 1-Zn-PPA and 1-NLG and their proposed coassembly into 1-NPs for sonophotodynamic immunotherapy.^138^ (b) A GSH-activated sonosensitizer prodrug was selectively activated at tumor sites for switch-on SDT activity and fluorescence.^139^ Adapted with permission from refs (138 and 139). Copyright 2023, John Wiley and Sons. Copyright 2023, John Wiley and Sons.

Triggered activation of sensitizers is another strategy shared by PDT and SDT, as exemplified by recent publications from the groups of Ye^138^ and An.^139^ Ye elegantly designed a self-assembled glutathione (GSH)-activatable nanosensitizer 1-NPs (Figure 14a), composed of two GSH-reducible amphiphilic probes (1-Zn-PPA and 1-NLG).^138^ In GSH-abundant tumor microenvironments, 1-NPs activate to release Zn-PPA-SH. This compound could further bind endogenous albumin, allowing to (1) turn on the fluorescence of AO-Luc at 547 nm and of Zn-PPA at 672 nm, and (2) switch on the SDT and PDT activities. Moreover, NLG919 is released concurrently, resulting in IDO1 inhibition and reducing tumor-associated immunosuppression. Further, this reduction process modifies the longitudinal relaxivity of the Gd-DOTA, serving as a magnetic resonance imaging contrast and enabling the monitoring of the GSH-triggered disassembly process. After systemic injection in vivo, 1-NPs are effective for bimodal fluorescence and magnetic resonance imaging-guided sono-photodynamic immunotherapy of orthotropic breast and brain tumors in mice under combined ultrasound (1 MHz) and 671 nm laser irradiation.

An’s design features a tetrahydroxy porphyrin sonosensitizing unit covalently bonded to a GSH-responsive quencher moiety (Figure 14b).^139^ In its inactive form, this sensitizer-prodrug shows limited fluorescence and a low capacity for ROS generation under ultrasound irradiation (30 kHz). However, it can be activated by GSH to release tetrahydroxy porphyrin. This release ’turns on’ both the fluorescence and sonosensitizing activity of the compound. Due to the overexpression of GSH in tumor tissues, this prodrug strategy enables selective activation of SDT in tumors, reducing the potential for adverse effects on the surrounding healthy tissue.

### 3.6 Bioreductive Prodrug Activation under SDT-Induced Hypoxia

**Figure 15:**
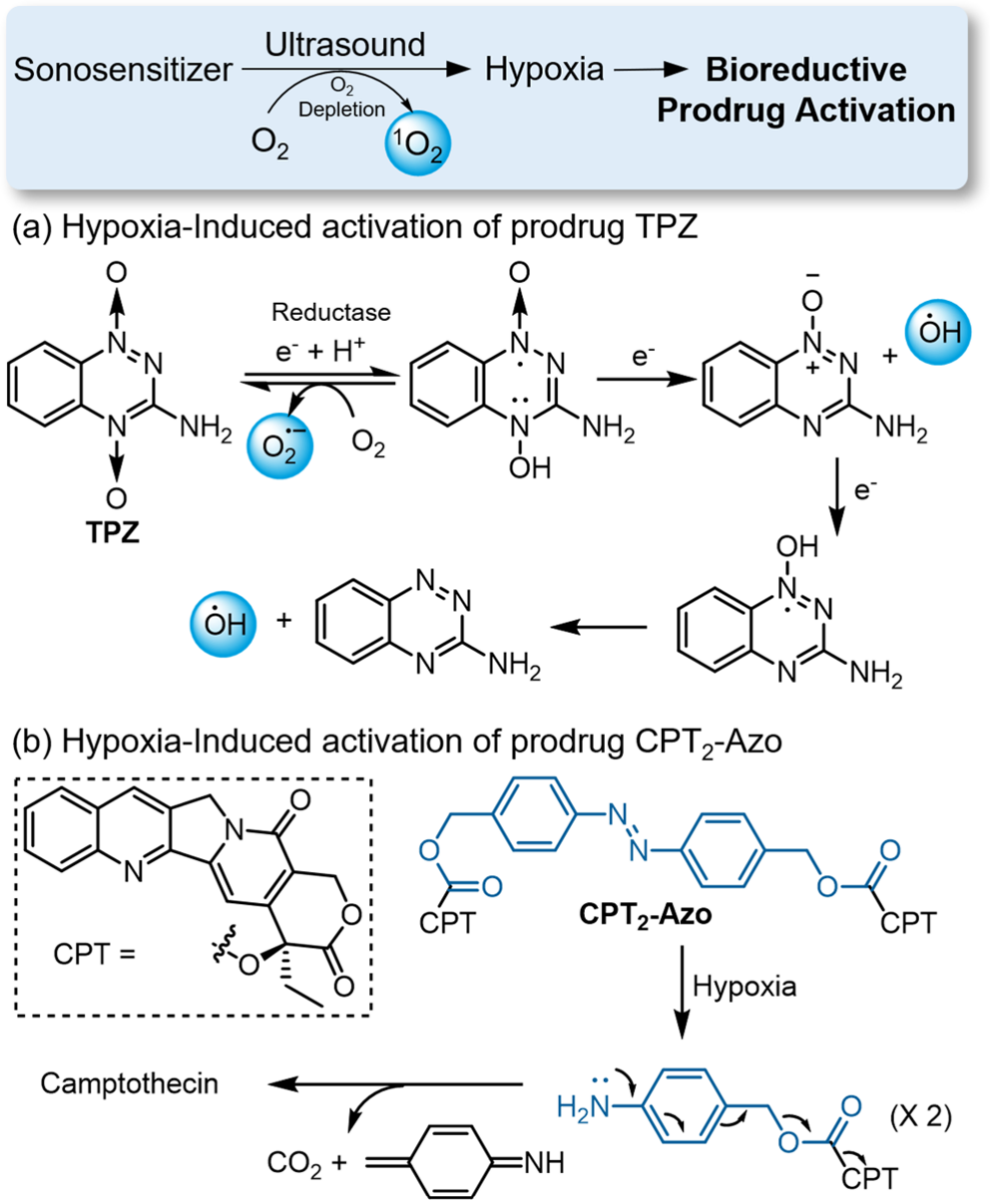
SDT treatments induced hypoxic environments and activated bioreductive prodrugs TPZ (a) and CPT_2_-Azo (b).

Another general strategy for controlling prodrug activation with ultrasound involves the induction of a hypoxic environment by SDT and utilizing the hypoxic bioreductive environment to activate redox-responsive prodrugs. For example, Zhang and Hou developed a TPZ/HMTNPs-SNO nanosystem (Figure 15a) by loading the bioreductive prodrug tirapazamine (TPZ) into S-nitrosothiol (R-SNO)-modified hollow mesoporous titanium dioxide nanoparticles (HMTNPs).^140^ The HMTNPs function as sonosensitizers to generate ROS under ultrasound irradiation (1 MHz), consuming oxygen in tumor and creating a hypoxic environment. Subsequently, TPZ undergoes a one-electron reduction to form a radical anion, which is further converted into either a hydroxyl radical or an oxidizing radical, ultimately resulting in DNA damage. Concurrently, the generated ROS sensitizes the -SNO groups to release nitric oxide (NO), synergistically improving the anticancer efficacy of SDT. Additionally, the echogenic property of NO endows this nanoplatform as a contrast agent for ultrasound imaging.

Huang and co-workers developed a metal–organic framework(MOF)-based nanoplatform (Figure 15b), integrating a hypoxia-activatable CPT prodrug CPT_2_-Azo with a porphyrin sonosensitizer TCPP, which are immobilized into the mesopores of the MOFs.^141^ The crystalline structures of these MOFs effectively incorporate small-molecule cargos, overcoming challenges such as self-quenching of sensitizers and the rapid diffusion of ^1^O_2_. After efficient endocytosis by tumor cells, this nanocomplex produces singlet oxygen under ultrasound stimulation, inducing apoptotic cell death while simultaneously aggravating the hypoxic conditions. This exacerbation of hypoxia triggers a more efficient bioreduction of CPT_2_-Azo prodrug, releasing the active form of CPT, thereby enhancing the therapeutic impact.

### 3.7 Noninvasive Optogenetic Activation by Ultrasound-Mediated Chemiluminescence

**Figure 16:**
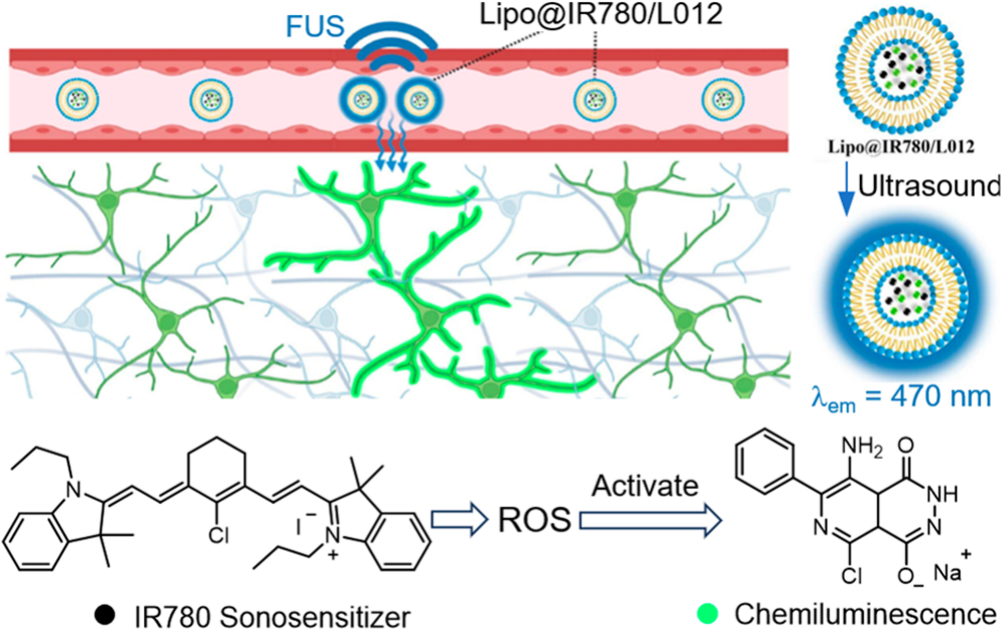
Ultrasound-triggered blue light emission by Lipo@IR780/L012 nanoparticles was harnessed for optogenetic stimulation of opsin-expressing neurons. The mechanism for ultrasound-induced chemiluminescence is illustrated at the bottom. Adapted with permission from ref (52). Copyright 2023, America Chemical Society.

Related to the prodrug-activation concept, ultrasound-mediated optogenetics is a promising research direction.^142^ Wang and Hong introduced a remarkable ultrasound-controlled optogenetic system (Figure 16), which could potentially be adapted for activating various functions for therapeutic applications. In their design, a chemiluminescent compound, L012, and a sonosensitizer, IR780, are encapsulated within lipid nanoparticles to form a nanoparticle-based light source that is controllable by focused ultrasound (1.5 MHz).^52^ When exposed to ultrasound, the IR780 generates ROS which in turn activate L012, leading to chemiluminescence. This light emission is then harnessed to activate opsin, a light-sensitive protein expressed by neurons. Their study demonstrated that these Lipo@IR780/L012 nanoparticles can emit blue light under FUS, which activates the CheRiff-expressing spiking HEK cells. Furthermore, in vivo experiments demonstrated that motor cortex neurons in Thy1-ChR2-YFP transgenic mice could be temporarily and reversibly activated via repetitive FUS irradiation after systemic administration of Lipo@IR780/L012, thus achieving noninvasive sono-optogenetic brain stimulation. We note that the ultrasound-induced, ROS-mediated chemiluminescence reported in this study is fundamentally different as compared to ultrasound-induced light emission from the mechanochemical activation of dioxetane mechanophores.^34,143^

## 4 Other Strategies for Ultrasound-Mediated Prodrug Activation

In this section, we briefly overview the inherent therapeutic bioeffects of ultrasound in the absence of sonosensitizers, and highlight a few non-SDT strategies where the prodrug activation does not rely on the presence of a sonosensitizer. It is worth noting, however, that the approaches discussed in sections 4.2 and 4.3 could also be regarded as SDT systems, where the prodrug structures themselves act as sensitizers. The therapeutic effects of ultrasound include: (1) the energy carried by ultrasound can convert into thermal energy through mechanical friction, facilitating deep-tissue thermal ablation^144^ (rapid temperature increase to >60 °C causing coagulative necrosis) or hyperthermia^145−147^ (raising tissue temperatures to 40–45 °C for various durations). (2) Collapsing cavitations can also exert sonomechanical effects that can cause histototripsy^147−149^ (cavitation implosion mechanically disrupts tissue structures without significant thermal effects), sonoporation^150,151^ (i.e., ultrasound-induced formation of transient pores in cell membrane enhances membrane permeability), and enhanced extravasation.^152,153^ (3) Additionally, as discussed in Section 3, acoustic cavitation alone can induce pyrolysis and enable high-energy reactions to produce therapeutic species such as cytotoxic radicals.

### 4.1 Liberation of Endogenous Enzyme Triggers Prodrug Activation

Lammers and co-workers developed an “enzyme-directed prodrug therapy” strategy that we summarize into two key steps:^154^ First, HIFU-induced (1.3 MHz) mechanical cell ablation in the targeted sites leads to the release of β-glucuronidase (β-GUS), an enzyme that is typically confined within cells under physiological conditions and found specifically in lysosomes; Second, the cell lysate containing β-GUS then enzymatically catalyzes the activation of β-d-glucuronide-capped doxorubicin prodrugs. Deckers reported a similar system combining a DOX-glucuronide prodrug nanogel and the HIFU-induced (1.3 MHz) liberation of endogenous β-GUS from cells.^155^

### 4.2 Sonoluminescence-Mediated Drug Activation

**Scheme 2:**
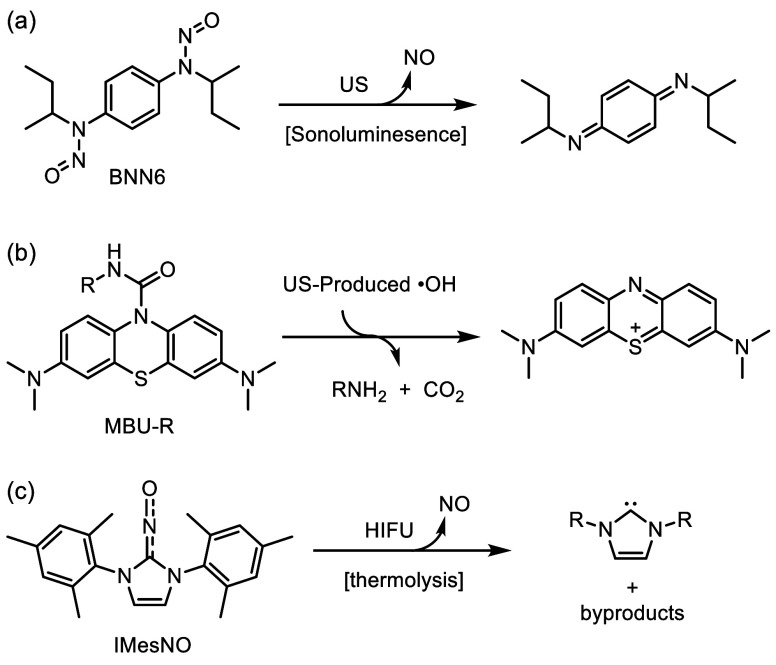
(a) Ultrasound-Responsive Prodrug BNN6 Releases Nitric Oxide through Proposed Sonoluminescence-Mediated Photochemical Activation.^156,157^ (b) Urea-Based Prodrug MBU-R Activated by Hydroxy Radicals Generated through Acoustic Cavitation, Leading to Release of Amine-Based Drug Molecule along with Methylene Blue and CO_2_.^159^ (c) IMesNO Prodrug Releases NO via HIFU-Mediated thermolysis (local temperature increase)^160^

He and co-workers discovered BNN6 (Scheme 2a), an ultrasound-responsive prodrug that releases nitric oxide (NO) through proposed sonoluminescence-mediated photochemical activation.^156,157^ Kim and co-workers combined BNN6 with piezoelectric barium titanate nanoparticle (BTNP) coated with polydopamine.^158^ Upon systemic administration and targeted ultrasound application (plane wave or HIFU, 1 or 1.5 or 2.2 MHz), these nanoparticles release NO near the target site, transiently opening the blood-brain barrier (BBB) and enabling nanoparticle accumulation in the brain. It was experimentally confirmed that NO release plays a critical role in disrupting the tight junction of the BBB and accumulation of nanoparticles in the brain. Under ultrasound, the nanoparticles generate a direct current that stimulates dopamine release from neuron-like cells. In a Parkinson’s disease mouse model, this treatment alleviated symptoms without significant toxicity.

### 4.3 Pyrolysis/Thermolysis-Mediated Drug Activation

Peng and co-workers elegantly designed urea-bond-containing prodrugs based on methylene blue (Scheme 2b).^159^ Upon sonication with therapeutic ultrasound (1 MHz), the urea bonds linked with primary amines are selectively cleaved under the ultrasound-induced oxidative environment (•OH radicals) around the cavitation bubble-liquid interface, releasing free methylene blue accompanied by recovered absorbance, fluorescence, and photosensitivity. Moreover, an FDA-approved alkylating agent (i.e., melphalan) bearing urea bond is also developed (MBU-Mel), successfully achieving ultrasound-triggered drug release in deep-seated cancer cells (mimic with 1 cm pigskin).

Kim and co-workers developed a different type of NO-prodrug (IMesNO) that releases NO via HIFU-mediated (1.5 MHz) thermolysis (Scheme 2c).^160^ They employed block copolymer-based micelles to encapsulate this hydrophobic IMesNO along with DOX, targeting their delivery to tumors via the enhanced permeability and retention (EPR) effect. Upon the application of HIFU, the IMesNO released NO, which enhanced the EPR effect within the tumor tissue. This enhancement significantly accelerated the accumulation of DOX in the tumor, leading to improved antitumor therapeutic efficacy. Additionally, Chen delivered l-arginine as an ultrasound-responsive NO prodrug, which is oxidized by ultrasound-produced ROS to generate NO for cancer treatment.^161^

## 5 Conclusion and Outlook

Ultrasound has emerged as a significant tool in medical applications, particularly due to its capability for noninvasive, deep-tissue penetration. The past five years have seen notable advancements in ultrasound-responsive prodrug systems, primarily involving two mechanistic strategies: polymer mechanochemistry (Section 2) and sonodynamic therapy (Section 3). Further, Section 4 discusses additional ultrasound-based methods beyond sonomechanical or sonodynamic effects. Cavitation is responsible for both mechanochemical and sonodynamic activation of prodrugs, and mechanochemical transformations and sonosensitizer activation are not a result of direct interaction between sound waves and materials.

The field of ultrasound-responsive prodrug strategies is rapidly evolving, presenting numerous research and discovery opportunities. Despite substantial advancements, further research is essential to fully understand the interaction between ultrasound of varying parameters and synthetic drug delivery systems and biological entities. In polymer mechanochemistry, it is important to enable mechanochemical activation of prodrug structures under mild, biocompatible ultrasound conditions through innovations that more effectively couple ultrasonic mechanical waves with mechanophores,^33,48^ or through the development of highly mechanosensitive structures.^162,163^ In SDT, exploring new ultrasound-mediated chemistries for prodrug activation, such as ROS-cleavage chemistry, redox and organic radical chemistries, could greatly diversify sonodynamically controlled prodrug strategies.

Additionally, the integration of ultrasound-responsive prodrug research with emerging therapeutic approaches, such as sonogenetics^142^ and combination immunotherapies,^126^ offers another promising avenue for exploration. Crucially, interdisciplinary collaboration across organic chemistry, materials science, biomedical engineering, and clinical practice is essential for translating these innovative concepts into practical medical advancements.
